# Cytomorphology review of 100 newly diagnosed lower-risk MDS patients in the European LeukemiaNet MDS (EUMDS) registry reveals a high inter-observer concordance

**DOI:** 10.1007/s00277-017-3009-7

**Published:** 2017-05-20

**Authors:** Louise de Swart, Alex Smith, Marius MacKenzie, Argiris Symeonidis, Judith Neukirchen, Dana Mikulenková, Teresa Vallespí, Gina Zini, Malgorzata Paszkowska-Kowalewska, Anton Kruger, Leonie Saft, Pierre Fenaux, David Bowen, Eva Hellström-Lindberg, Jaroslav Čermák, Reinhard Stauder, Aurelia Tatic, Mette Skov Holm, Luca Malcovati, Krzysztof Mądry, Jackie Droste, Nicole Blijlevens, Theo de Witte, Ulrich Germing

**Affiliations:** 1 Department of Haematology, Radboud University Medical Centre, Nijmegen, The Netherlands; 2 Epidemiology and Cancer Statistics Group, Department of Health Sciences, University of York, York, UK; 3 Department of Medicine, Division of Haematology, University of Patras Medical School, Patras, Greece; 4 Department of Haematology, Oncology and Clinical Immunology, Universitätsklinik Düsseldorf, Düsseldorf, Germany; 5 Department of Clinical Haematology, Institution of Haematology and Blood Transfusion, Praha, Czech Republic; 6 Department of Haematology, Haematological Cytogenetics Unit, University Hospital of Vall d’Hebron, Barcelona, Spain; 7 Haematology Institute, Catholic University of Sacred Heart, Rome, Italy; 8 Department of Haematology, Oncology and Internal Medicine, Warszawa Medical University, Warszawa, Poland; 9 Department of Haematology, Royal Cornwall Hospital, Truro, UK; 10 Department of Pathology and Cytology, Karolinska University Hospital and Karolinska Institutet, Stockholm, Sweden; 11 Service d’Hématologie, Hôpital Saint-Louis, Assistance Publique des Hôpitaux de Paris (AP-HP) and Université Paris 7, Paris, France; 12 St. James’s Institute of Oncology, Leeds Teaching Hospitals, Leeds, UK; 13 Department of Medicine, Division of Haematology, Karolinska Institutet, Stockholm, Sweden; 14 Department of Internal Medicine V (Haematology and Oncology), Innsbruck Medical University, Innsbruck, Austria; 15 Centre of Haematology and Bone Marrow Transplantation, Fundeni Clinical Institute, Bucharest, Romania; 16 Department of Haematology, Aarhus University Hospital, Aarhus, Denmark; 17 Department of Haematology Oncology, Fondazione IRCCS Policlinico San Matteo, University of Pavia, Pavia, Italy; 18 Department of Tumor Immunology - Nijmegen Centre for Molecular Life Sciences, Radboud University Medical Centre (TIL, 278), P.O. Box 9101, 6500 HB Nijmegen, the Netherlands

**Keywords:** Myelodysplastic syndromes (MDS), Diagnostics, Cytomorphology review, Inter-observer variability

## Abstract

The European LeukemiaNet MDS (EUMDS) registry is collecting data of myelodysplastic syndrome (MDS) patients belonging to the IPSS low or intermediate-1 category, newly diagnosed by local cytologists. The diagnosis of MDS can be challenging, and some data report inter-observer variability with regard to the assessment of the MDS subtype. In order to ensure that correct diagnoses were made by the participating centres, blood and bone marrow slides of 10% of the first 1000 patients were reviewed by an 11-person panel of cytomorphologists. All slides were rated by at least 3 panel members (median 8 panel members; range 3–9). Marrow slides from 98 out of 105 patients were of good quality and therefore could be rated properly according to the WHO 2001 classification, including assessment of dysplastic lineages. The agreement between the reviewers whether the diagnosis was MDS or non-MDS was strong with an intra-class correlation coefficient (ICC) of 0.85. Six cases were detected not to fit the entry criteria of the registry, because they were diagnosed uniformly as CMML or AML by the panel members. The agreement by WHO 2001 classification was strong as well (ICC = 0.83). The concordance of the assessment of dysplastic lineages was substantial for megakaryopoiesis and myelopoiesis and moderate for erythropoiesis. Our data show that in general, the inter-observer agreement was high and a very low percentage of misdiagnosed cases had been entered into the EUMDS registry. Further studies including histomorphology are warranted.

## Introduction

Myelodysplastic syndromes (MDS) are a heterogeneous group of clonal diseases, characterized by cytopenias and dysplastic features in blood and bone marrow. Due to the heterogeneity of clinical presentation, degree of dysplastic features and a considerable list of differential diagnosis, the diagnosis of MDS is often a challenge, particularly in lower-risk MDS. The diagnosis of MDS is primarily based on cytomorphologic and histopathologic assessment of dysplasia, but cytogenetic evaluation is crucial for the classification and to determine the risk assessment using the revised International Prognostic Scoring System (IPSS-R) score [1]. Flow cytometry and molecular analysis provide additional valuable information to refine the diagnosis and prognosis of MDS, but the clinical and therapeutic impact of these methods is under investigation [2, 3]. To make a correct diagnosis of MDS, good cytomorphologic experience and adequate clinical information are required, including concomitant medication, concurrent infections and comorbidity, and other possible causes of cytopenia or dysplastic features. Therefore, a multidisciplinary approach is often required to make the definite diagnosis of MDS and to exclude the possibility of other disorders mimicking MDS [4, 5].

In the prospective European LeukemiaNet MDS (EUMDS) registry, patients with a newly diagnosed MDS belonging to the low or intermediate-1 IPSS risk group are included, within 100 days of making the definite diagnosis of MDS [6]. Patients with higher-risk MDS or therapy-related MDS are excluded. National and local ethics committees have approved the study, and all patients provided written informed consent for inclusion in the registry. The first 1000 patients were diagnosed with MDS between December 2007 and December 2010, and therefore, the diagnosis was based on the World Health Organization (WHO) 2001 classification, without central revision of diagnosis. Since a correct MDS diagnosis and classification are crucial in the EUMDS registry and may be difficult in the lower-risk group of MDS, a morphology sub-study was performed by reviewing 100 randomly selected cases by a panel group of 11 MDS cytomorphologists from 9 different countries. The cytomorphology sub-study was primarily aimed at a validation and reproducibility of the WHO 2001 criteria for MDS by an international panel and at the validation of original cytomorphologic accuracy and correct diagnostic inclusion into the registry. The aims of the study were (i) quality assessment of cytomorphologic diagnoses in the EUMDS registry and (ii) assessment of inter-observer variability in the review panel regarding diagnosis and degree of dysplasia.

## Design and methods

The cytomorphology review was performed by a panel comprising 11 morphologists during a 3-day meeting at the Heinrich Heine University medical Centre in Düsseldorf, Germany. The aim was to review the original diagnosis at time of inclusion in 10% of the patients in the EUMDS registry, with five additional cases to replace inadequate material (only bone marrow trephine biopsy or slides with poor quality unable to examine).

### Selection of cases

A total of 105 cases, representing all participating countries, were randomly selected by the Epidemiology and Cancer Statistics Group (ECSG) of the University of York who manages the Web-based EUMDS database. The distribution of the selected samples was dependent on the number of included patients in each country. The anonymization of the slides was done in Nijmegen at the Trial Office Haematology (TOH) of the Department of Haematology of the Radboud university medical centre (Radboudumc) before transporting the slides to Düsseldorf. The demographic, hematologic and prognostic characteristics of the subjects included in the morphology study were generally representative of the subjects included in the registry at the time of sampling [6].

### Clinical and haematologic characteristics

The review panel was provided with the following clinical and haematological characteristics of the patients under review: ID number (anonymous), date of diagnosis, haemoglobin (Hb), white blood cells (WBC), platelets, absolute neutrophil count (ANC), monocytes and IPSS cytogenetics category (good or intermediate-1 risk, but not information on del(5q)) at diagnosis. No original morphology report or previous diagnosis was provided to any of the panel members.

### Handling of slides

A minimum of 1 stained or unstained bone marrow slide, in most cases 1 bone marrow iron staining and 1 blood film, had been sent to the TOH in Nijmegen. All slides had to be from the date of diagnosis, preferably the identical slides on which the original diagnosis was made. Twenty unstained slides were stained at the Radboudumc using the May-Grünwald-Giemsa stain. The number of available iron stained slides was restricted to 45 patients, and in 11 patients, the percentage of ring sideroblasts were reported to be >14 by the centres. For these 11 patients, the original iron staining was accepted by the expert panel.

### Morphology review session

The 11 panel members reviewed the slides independently from each other during three consecutive days at the University of Düsseldorf. Each panel member used a separate Zeiss Axiostar Plus Transmitted-Light Microscope, provided by Zeiss, Germany. The percentage of blasts in blood and marrow were counted on the basis of 300 nucleated cells precisely by at least 3 experts during the review procedure; the other panel members estimated the blast count divided in categories (<5%, 5–9% or ≥10% blasts). The degree of dysplasia in all cell lines was scored semi-quantitatively by each reviewer, divided into three categories (absent: <10% dysplastic cells, mild or obvious dysplasia). All slides were reviewed by at least three individual panel members. Following individual slide review, the full panel met to consider characteristics and diagnostic problems of specific cases. Diagnoses were made according to the WHO classifications of 2001 [7, 8].

Definition of the review diagnosis:

1. WHO 2001 Definite diagnosis: MDS diagnosis of each panel member based on consensus blast count done by the whole group of that specific case and scored dysplasia in the erythropoiesis, myelopoiesis and megakaryopoiesis.
2. WHO 2001 Expert diagnosis: MDS diagnosis of each panel member based on individual blast count (counted or estimated) and MDS diagnosis in conclusion.

### Statistical analysis

WHO 2001 diagnoses recorded by the panel members were categorized into the following groups:

1. Refractory anaemia (RA) and refractory anaemia with ringed sideroblasts (RARS),
2. Refractory cytopenia with multilineage dysplasia (RCMD) and refractory cytopenia with multilineage dysplasia and ringed sideroblasts (RCMD-RS),
3. Refractory anaemia with excess blasts-1 (RAEB-1),
4. Refractory anaemia with excess blasts-2/acute myeloid leukaemia (RAEB-2/AML)
5. 5q-syndrome
6. Chronic myelomonocytic leukaemia (CMML)
7. Possible MDS (not definite MDS diagnosis)

All analyses were undertaken in Stata version 14; agreement between the 11 raters was measured using the intra-class correlation coefficient (ICC) calculated using the icc23 command [9]. The average ICC for the reliability of different reviewers was reported; ICC can range from 0 to 1, where 1 represents perfect agreement; agreement can be interpreted using the following categories proposed by Landis and Koch: <0 poor, 0.01–0.20 slight, 0.21–0.40 fair, 0.41–0.60 moderate, 0.61–0.80 substantial and 0.81–1.00 as almost perfect agreement [10].

## Results

Median age of the randomly selected patients at diagnosis was 72 years (range 21 to 95), there was a male predominance (67%) and the most common diagnosis recorded was RCMD (Table 1). In total, 774 slides were reviewed by the 11 raters, 45 slides (5.8%) were excluded from subsequent analyses as the panel members were unable to review the diagnosis due to the poor quality of the slide. One case was removed as only two panel members had reviewed it. Accordingly, in total, 98 cases were included in the review, and, on average, each case was reviewed by eight different panel members (range 3–9). In total, 727 diagnoses were made by the panel members.

**Table 1:** Patient characteristics at registration to the registry

	*N* (%)
Total	98 (100)
Age at diagnosis (years), median (range)	72.0 (21.0–95.0)
Sex	
Males	66 (67.3)
Females	32 (32.7)
Country	
Austria	2 (2.0)
Czech Republic	7 (7.1)
Denmark	3 (3.1)
France	30 (30.6)
Germany	7 (7.1)
Greece	11 (11.2)
Italy	2 (2.0)
Netherlands	8 (8.2)
Poland	2 (2.0)
Romania	4 (4.1)
Sweden	8 (8.2)
United Kingdom	14 (14.3)
Haemoglobin (g/dL), mean (SD)	10.5 (2.0)
WBC (10^9^/L) mean (SD)	4.9 (2.4)
Absolute neutrophil count (10^9^/L) mean (SD)	3.0 (2.0)
Platelets (10^9^/l) mean (SD)	183 (121)
WHO diagnosis provided at time of entry into the registry	
RA	17 (17.3)
RARS	20 (20.4)
RCMD	40 (40.8)
RCMD-RS	3 (3.1)
RAEB-1	14 (14.3)
5q-syndrome	4 (4.1)
IPSS Score	
Low	344 (47.3)
Intermediate-1	345 (47.5)
Not known	38 (5.2)

*RA* refractory anaemia, *RARS* refractory anaemia with ringed sideroblasts, *RCMD* Refractory cytopenia with multilineage dysplasia, *RCMD-RS* refractory cytopenia with multilineage dysplasia and ringed sideroblasts, *RAEB-1* refractory anaemia with excess blasts-1, 5q-syndrome

### Concordance of MDS diagnoses

**Fig. 1:**
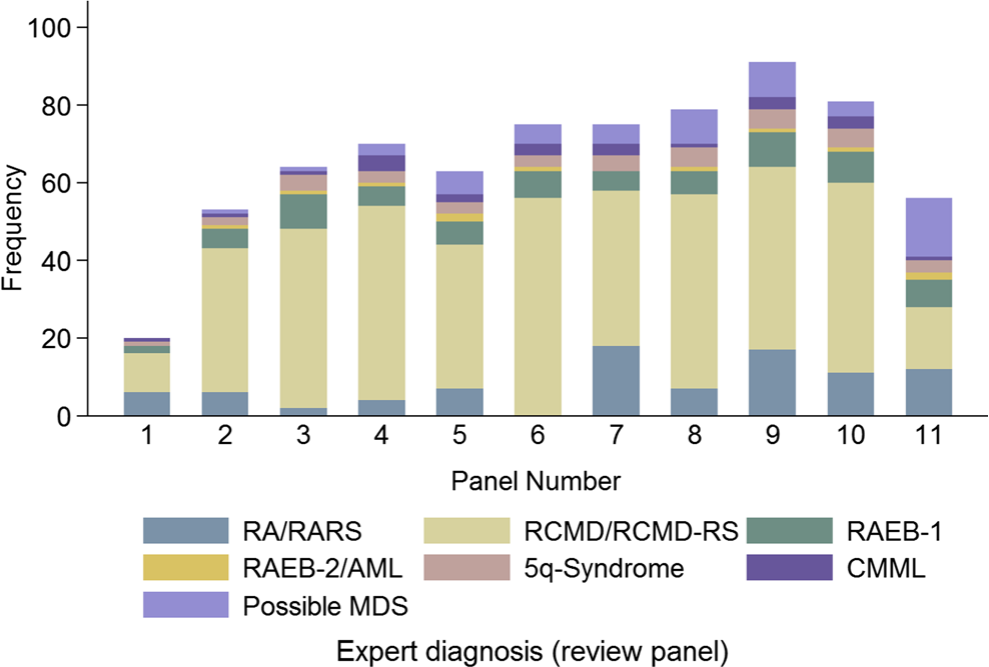
Proportion of cases reviewed by each panel member and expert diagnoses made

In a first step, the concordance with regard to the diagnosis of an MDS versus non-MDS was analysed. Differences were seen by reviewer in the proportion of cases reviewed, as can be seen in Fig. 1; in addition, this figure demonstrates the difference in the proportion of diagnoses queried to whether or not the case had MDS (‘possible MDS’). Overall, 8% of cases were classified as ‘possible’ MDS, but reviewer 11 classified 27% of cases to this category, all of them were rated to have <5% medullary blasts. For the majority of slides, a MDS diagnosis was made (88%). Eleven slides were classified as RAEB-2 or AML, 23 as CMML and 58 as possible MDS cases. In Table 2, the number of possible MDS is not included itself, but can be deducted from the numbers of different MDS diagnosis by panel review and the total number of slides (Table 2). Agreement between the reviewers whether the diagnosis was MDS or non-MDS was strong ICC = 0.85 (95% confidence intervals (95%CI): 0.80–0.89).

**Table 2:** Expert diagnoses (review panel) by original diagnoses by local cytologists (clinical)

	Original diagnosis by local cytologists				
Review panel diagnosis	RA/RARS (*n* = 37 pts)	RCMD/RCMD-RS (43 pts)	RAEB-1 (*n* = 14 pts)	5q-syndrome (*n* = 4 pts)	Total (*n* = 98 pts)
Total	265 (100.0)	326 (100.0)	106 (100.0)	30 (100.0)	727 (100.0)
RA/RARS	50 (18.9)	36 (11.0)	4 (3.8)	-	90 (12.4)
RCMD/RCMD-RS	155 (58.5)	232 (71.2)	51 (48.1)	-	438 (60.2)
RAEB-1	21 (7.9)	27 (8.3)	21 (19.8)	-	69 (9.5)
RAEB-2/AML	-	4 (1.2)	7 (6.6)	-	11 (1.5)
5q-syndrome	-	-	8 (7.5)	30 (100.0)	38 (5.2)
CMML	9 (3.4)	-	14 (13.2)	-	23 (3.2)

*RA* refractory anaemia, *RARS* refractory anaemia with ringed sideroblasts, *RCMD* refractory cytopenia with multilineage dysplasia, *RCMD-RS* refractory cytopenia with multilineage dysplasia and ringed sideroblast, *RAEB-1* refractory anaemia with excess blasts-1, *RAEB-2* refractory anaemia with excess blasts, *AML* acute myeloid leukaemia, 5q-syndrome, *CMML* chronic myelomonocytic leukaemia, *pts* patients

### Concordance of WHO 2001 diagnoses

**Fig. 2:**
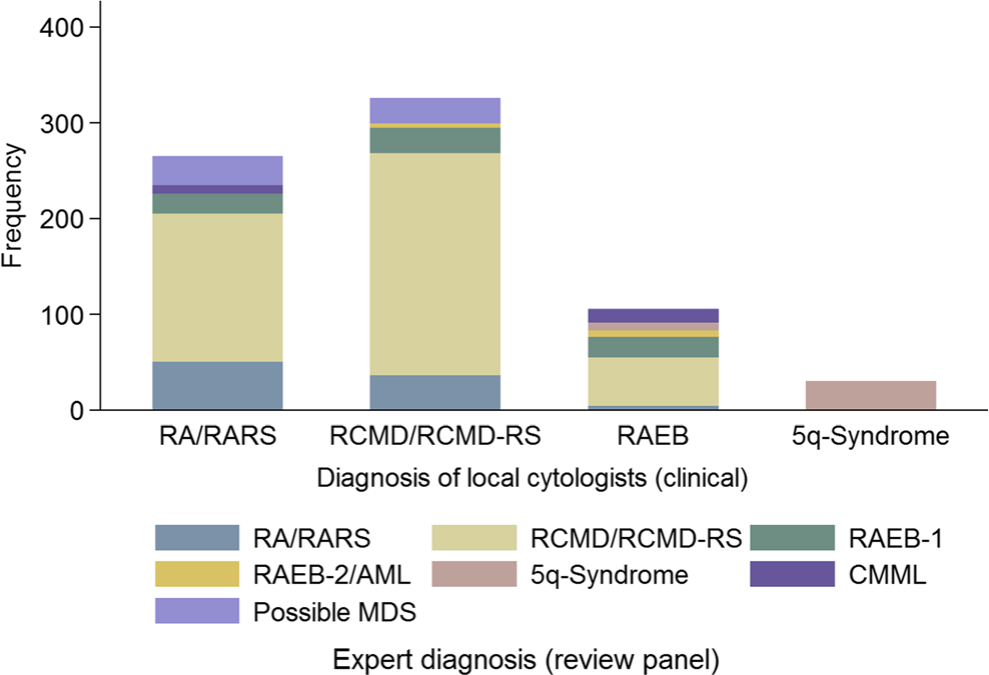
Original diagnoses by local cytologists versus expert diagnoses

In the next step, the concordance of WHO 2001 subtypes was analysed (Fig. 2). The agreement by WHO 2001 diagnoses was almost identical; ICC = 0.83 (95%CI: 0.77–0.87). Table 2 describes the review panel diagnoses made versus the original diagnosis recorded in the registry. We also assessed the medullary blast categories by separating patients with <5%, 5–9%, 10–19% and >20% medullary blasts. A total of 88.4% of the slides were scored as less than 5% medullary blasts, 10% as RAEB-1, 0.9% as RAEB-2 and 0.5% as AML. The inter-observer variability for this categorization was very low, as only 6% of the slides were described differently by at least one panel member.

### Concordance of assessment of dysplasia

In addition, we analysed the concordance of the assessment of dysplastic lineages (Table 3). The number of slides in which either dyserythropoiesis (9.9%), dysmyelopoiesis (7.5%) or dysmegakaryopoiesis (21.1%) could not be assessed properly was relatively high, especially in the slides that were described as possible MDS. Looking at the dysplastic lineages in the different WHO types, it became obvious that in the unilineage dysplastic types (RA/RARS), in 23.8 and 9.4% of the slides, dysmyelopoiesis and dysmegakaryopoiesis were described, respectively, whereas in the RCMD/RCMD-RS group, these numbers were higher (49.5 and 54.9%). Dyserythropoiesis was described to be less frequent in the RA/RARS and 5q-syndrome groups as compared to the RCMD/RCMD-RS group. As expected, the highest percentage of dysmegakaryopoiesis was described in the 5q-syndrome group. In cases that were labelled ‘possible MDS’, the percentage of dyserythropoiesis was lower compared to definite MDS cases, and only a very small proportion showed dysmyelopoiesis with none showing dysmegakaryopoiesis. The agreement of the reviewers with regard to the assessment of dysmegakaryopoiesis (0.60; 95% CI: 0.48, 0.71) and dysmyelopoiesis (0.72; 95% CI: 0.64, 0.80) was substantial and for dyserythropoiesis was moderate, close to substantial (0.591; 95% CI: 0.464, 0.699).

**Table 3:** Proportion with abnormalities by the expert diagnosis

	RA/RARS	RCMD/RCMD-RS	RAEB-1	RAEB-2/AML	5q-syndrome	CMML	Possible MDS	Total
Total number (%)	90 (100)	438 (100)	69 (100)	11 (100)	38 (100)	23 (100)	58 (100)	727 (100)
Dyserythropoiesis								
No	19 (25.7)	14 (3.3)	10 (16.7)	-	3 (9.1)	3 (13.6)	13 (41.9)	62 (9.5)
Mild	29 (39.2)	159 (37.2)	26 (43.3)	4 (50.0)	17 (51.5)	11 (50.0)	16 (51.6)	262 (40.0)
Obvious	26 (35.1)	254 (59.5)	24 (40.0)	4 (50.0)	13 (39.4)	8 (36.4)	2 (6.5)	331 (50.5)
Not known	16	11	9	3	5	1	27	72
Dysgranulopoiesis								
No	53 (66.3)	43 (10.1)	6 (9.2)	-	9 (25.7)	5 (22.7)	22 (62.9)	138 (20.5)
Mild	8 (10.0)	172 (40.4)	20 (30.8)	4 (44.4)	13 (37.1)	7 (31.8)	12 (34.3)	236 (35.1)
Obvious	19 (23.8)	211 (49.5)	39 (60.0)	5 (55.6)	13 (37.1)	10 (45.5)	1 (2.9)	298 (44.3)
Not known	10	12	4	2	3	1	23	55
Dysmegakaryopoiesis								
No	42 (79.2)	45 (12.2)	14 (24.6)	-	1 (2.7)	7 (33.3)	20 (71.4)	129 (22.5)
Mild	6 (11.3)	122 (33.0)	19 (33.3)	3 (42.9)	5 (13.5)	5 (23.8)	8 (28.6)	168 (29.3)
Obvious	5 (9.4)	203 (54.9)	24 (42.1)	4 (57.1)	31 (83.8)	9 (42.9)	-	276 (48.2)
Not known	37	68	12	4	1	2	30	154

### Comparison of original diagnoses and panel diagnoses

In a last step, we compared the diagnoses made by the centres that entered the patients into the lower-risk EUMDS registry. We identified 6 cases (6.1%) that did not fulfil the entry criteria. Four patients were described to have CMML by all panel members who assessed the slides of blood and marrow with 100% agreement. Both patients were originally entered into the registry as RA and RAEB-1. Two patients were diagnosed as RAEB-2 or AML by the panel members with 100% agreement. These patients were entered into the registry as RCMD and RAEB-1.

## Discussion

Diagnosis of MDS is still based primarily on cytomorphology of blood and marrow, accompanied by histomorphology, cytogenetics, molecular findings and flow cytometry. The EUMDS registry is collecting data of newly diagnosed lower-risk MDS patients. Eligibility for entry into the registry is based on the local assessment of cytomorphology at the participating centres in 14 different countries and 118 sites. In order to assess the quality and accuracy of the MDS diagnoses in the registry, we organized a working group reviewing 10% of the first 1000 patients that were included into the registry. In our cytomorphologic review of 100 newly diagnosed lower-risk MDS patients, we could clearly demonstrate a high accuracy of the original diagnoses at entry into the registry and a high inter-observer concordance of the panel members with regard to diagnosis of MDS and assessment of the lineage involvement of dysplasia.

An important prerequisite for cytology is satisfactory quality-stained slides, in our case well-prepared blood and marrow films and ‘bone marrow aspirate squash’ slides without many preparation artefacts. Only 6% of the slides were of poor quality and could not be assessed properly, whereas 94% could be validated. Cytomorphology in MDS can be challenging, even for experienced morphologists, and sometimes a definite diagnosis is not possible or only based on genetic findings [11]. Minimal diagnostic criteria for the diagnosis of an MDS cannot be based on morphology only, as no single sign of dysplasia is MDS specific [12]. The slides that were evaluated during our microscope session were blinded, and the panel member did not have information on the clinical course of the patients and were not biased in their judgement. We assembled a very heterogeneous panel of cytologists in terms of the morphological experience of the members, and therefore we were prepared to be faced with discrepancies in the assessment of the diagnosis. Surprisingly, most disagreements were not significant. First of all, a small number of slides were detected as CMML and RAEB-2 and AML cases. This task was the easiest for the panel members as there was no disagreement. As a result, only 6% of the patients who entered into the registry were misdiagnosed by the centres. This confirms that the data quality of the registry is high and that subsequent analyses based on diagnosis by the local cytomorphologists are likely to be valid. The assessment of medullary blast counts was also extremely uniform, as only 6% of the cases were differently categorized (<5% blasts versus 5–9% blasts).

The assessment of lineage involvement of dysplasia was less concordant. Inter-observer variability with regard to description of dysplasia was reported by several groups [13–15]. Our data show that our Panel team had substantial agreement on the assessment of dysmyelopoiesis and dysmegakaryopoiesis, whereas the assessment of dyserythropoiesis was less concordant. This finding is plausible, as some signs of dysplasia in myelo- and megakaryopoiesis are more specific for myeloid malignancies like micromegakaryocytes, mononuclear megakaryocytes, pseudo-pelger cells and degranulated myeloid precursors. These signs of dysplasia are usually not described in the marrow with reactive changes due to inflammation, toxic effects and others [12]. Morphological erythroid dysplasia can be evident in benign disorders like hemolysis, megaloblastic anemias and others. As a result, the panel members differed moderately in the categorization of uni- versus multilineage dysplasia. There is growing evidence that flow cytometry with regard to properly described dysplasia of erythropoiesis can be utilized if performed in a sophisticated way [3, 16, 17]. The separation of lower-risk MDS cases, namely distinguishing uni- and multilineage dysplasia, ring sideroblastic phenotype, and deletion of chromosome 5 is of great prognostic value, as shown by different groups [18–21]. As our data show, knowledge of cytomorphology is necessary to properly describe dysplasia and to use the classification systems accordingly. Therefore, skills in cytomorphology are still mandatory in centres where patients with MDS are diagnosed and treated accordingly [22, 23].

The WHO 2001 proposals for the classification of MDS took into account the separation of uni- and multilineage dysplasia within the non-blastic MDS cases with ring sideroblasts. This feature was omitted by the WHO 2008 [24] proposals but was reintroduced in the WHO 2016 classification [21]. As a result, our data generally reflect the WHO 2016 classification with the exception of minor changes of the definition of MDS del(5q) and MDS unclassified and the introduction of SF3B1 mutations as classifying elements.

The slides that were evaluated during our microscope sessions were blinded and the panel members did not have information on the clinical course of the disease. The only bias was the information that slides have been diagnosed as lower-risk MDS by the local cytologists.

Limitations of the project were some missing iron stains, not primarily affecting the morphological results with regard to eligibility of the patients into the registry. An even better assessment of morphology could have been reached by adding histomorphology for all cases. Although the impact of histomorphology in lower-risk MDS is limited as compared with cytomorphology, it can be helpful in cases with poor cytologic material and can help to properly assess the cellularity and fibrosis [25]. Therefore, another panel review, including histopathologists, could be of added value.

In summary, there was a high inter-observer agreement after the elimination of poor quality slides within our very heterogeneous panel of 11 morphologists. As a result, the inclusion of the patients into the EUMDS registry could be shown to be correct in the vast majority of the patients. The 6 patients originally not diagnosed correctly by the local cytologists have not been withdrawn from the registry. The registry is assessing patients who were diagnosed in the real world as lower-risk MDS and managed as such. Therefore, a central morphology review is not mandatory for this particular registry.
